# Minimally Invasive Therapies for the Management of Dental Caries—A Literature Review

**DOI:** 10.3390/dj9120147

**Published:** 2021-12-07

**Authors:** Hetal Desai, Cameron A. Stewart, Yoav Finer

**Affiliations:** 1 Faculty of Dentistry, University of Toronto, Toronto, ON M5G 1G6, Canada; hetal.desai@mail.utoronto.ca (H.D.); cameron.stewart@mail.utoronto.ca (C.A.S.); 2 Institute of Biomedical Engineering, University of Toronto, Toronto, ON M5S 3G9, Canada

**Keywords:** minimally invasive dentistry, caries management, dental restorative technique, resin infiltration, sealants, fluoride, cariology

## Abstract

In recent years, due to a better understanding of the caries pathology and advances in dental materials, the utilization of non-invasive and minimally invasive techniques that delay/obviate the need for traditional restorations has started gaining momentum. This literature review focuses on some of these approaches, including fluoride varnish, silver diamine fluoride, resin sealants, resin infiltration, chemomechanical caries removal and atraumatic restorative treatment, in the context of their chemistries, indications for use, clinical efficacy, factors determining efficacy and limitations. Additionally, we discuss strategies currently being explored to enhance the antimicrobial properties of these treatment modalities to expand the scope of their application.

## 1. Introduction

Traditional restorative techniques employed in the treatment of dental caries involve the removal of large amounts of tooth structure to (1) eliminate cariogenic bacteria to stop the decay process, (2) prepare the tooth to mechanically retain the restoration and withstand occlusal forces and (3) remove demineralized dentin [1]. However, the advent of adhesive and bioactive dental materials that micromechanically bond to the tooth and provide support, have made extensive removal of tooth structure for restoration retention unnecessary [1]. These materials also provide an excellent peripheral seal and isolate the carious lesion from the oral environment, facilitating caries arrest without complete decay excavation [2,3,4]. Recent studies suggest that demineralized but structurally intact dentin can be remineralized [5,6,7]. To preserve affected dentin that can be remineralized, attempts are made to selectively remove infected dentin from deep carious lesions during cavity preparation. Clinical studies have shown that this approach reduces the risk of pulp exposure [8] and increases the probability of treatment success fourfold relative to conventional excavation [9]. Thus, a better understanding of the caries pathology combined with newer dental materials and evidence gathered from clinical studies have paved the way for non-invasive and minimally invasive treatment approaches, which emphasize the maximum conservation of healthy tooth structure and avert the need for conventional restorations that often plunge the tooth into a treatment–retreatment cycle, often referred to as the “death spiral of restoration” [10].

Non-invasive treatments (e.g., flossing and fluoride application) focus on the reduction of the biofilm cariogenicity via plaque control and rely largely on patient compliance [11], whereas minimally invasive treatments (e.g., resin infiltration and sealants) involve the formation of a mechanical barrier to protect the tooth against the biofilm and are less dependent on patient compliance [12]. Specific indications for each of these techniques vary and their efficacy is governed by factors such as individual caries risk, lifestyle, extent of decay, tooth surface involved, number of surfaces involved and type of dentition. When used appropriately, these treatments yield results comparable to traditional treatment in terms of clinical outcomes and restoration longevity and have been found to be less time-consuming [13,14], associated with less dental anxiety and discomfort [15,16] and more cost-effective [14,17,18] in the long term. Therefore, it is imperative to use these techniques as the first line of treatment whenever possible, resorting to invasive restorative approaches only when these strategies are deemed to be insufficient for caries management. Lastly, the overall success of these techniques in preventing and arresting carious lesions provides impetus for the continued development of these approaches.

The following discussion focuses on the review of some non-invasive and minimally invasive approaches.

## 2. Methods

Relevant articles were retrieved from PubMed database using the following keywords either alone or in combination: “minimally invasive dentistry”, “fluoride varnish”, “resin sealants”, “resin infiltration”, “chemomechanical caries removal”, “atraumatic restorative treatment”, “caries arrest” and “caries management”. Systematic reviews and meta-analysis, literature reviews, randomized controlled trials and studies investigating strategies to improve the antimicrobial properties of the minimally invasive caries management techniques published in English language over the last 10 years were included in the review.

## 3. Chemical Management of Caries Using Fluoride Varnish

Fluoride varnishes (FV) are safe [19], do not cause permanent tooth discoloration, are well accepted by children [20] and can be easily applied by healthcare professionals [21]. They have been extensively, ubiquitously used to prevent dental caries; however, recent trials have shown reduced efficacy of FV in preventing dental decay. It is thought that this reduced effectiveness is because, in recent times, children are exposed to more fluoride sources, including water and toothpastes, than in the past, therefore, the effect of FV is not as prominent [22].

A recent meta-analysis showed that over a 2–3-year follow-up period, there was no statistical difference in the incidence of occlusal caries on first permanent molars following sealant and FV application [23]. These findings corroborate those of Chestnutt et al. who found that the effectiveness of FV in preventing occlusal decay on first permanent molars was higher but not statistically different from sealants (caries incidence 17.5% vs. 19.6%) following a 3-year period. However, FV was more cost-effective than sealant application [24]. For non-cavitated proximal lesions, meta-analysis results show that there is not enough evidence to support the use of FV to arrest decay. However, the effectiveness of a combination of 5% sodium fluoride and resin infiltration was more effective in arresting the progress of these lesions than resin infiltration alone [25]. Hence, it may be beneficial to use FV as adjunct therapy. Clinical trials evaluating the efficacy of SDF on caries arrest have consistently concluded that it outperforms fluoride varnish [26], and recent evidence-based ADA guidelines strongly recommend the use of SDF over fluoride varnish for the non-invasive treatment of cavitated carious lesion in primary teeth [27]. In preschoolers, it was previously thought that FV application could levy a protective effect and result in fewer caries-related hospitalizations. However, authors of a recent meta-analysis concluded that FV has only a “modest, uncertain” anti-caries effect in this age group [22] and, therefore, is not likely to contribute to a reduction in hospitalization rates.

## 4. Silver Diamine Fluoride

Silver Diamine Fluoride (SDF) is a liquid interim caries-arresting medicament that has been used to halt dental decay for decades in several countries including Japan, China, Argentina and Brazil [28,29,30,31]. In the U.S., SDF was approved for dentin desensitization in adults in 2014, and, since then, its off-label use in caries control has begun to gain widespread interest, especially amongst practitioners treating children [32]. Biannual application of 38% SDF has an 84.8% success rate in arresting caries [33], and its use to arrest cavitated lesions in primary teeth is supported by the American Academy of Pediatric Dentistry (AAPD) [34]. SDF is inexpensive [35], can be applied without the removal of infected soft dentin [36] and provides an effective treatment alternative in patients with behavioral issues and severe dental anxiety, medically fragile patients including those undergoing or having undergone radiation therapy and young pre-cooperative patients who need treatment under general anesthesia [16]. 

SDF contains silver and fluoride ions dissolved in an ammonia solution, which aids in the stabilization of silver fluoride. Thirty-eight% of SDF contains 44,800 ppm of fluoride ion, and although its exact mechanism of action is unknown, it is found to be effective in inhibiting the demineralization of carious lesions, facilitating remineralization and preventing the degradation of the dentinal organic matrix [37]. It can increase the mineral density of enamel lesions and microhardness of dentinal carious lesions [38,39]. Treatment with SDF has been shown to precipitate an insoluble surface layer of silver chloride, which acts as a protective layer and inhibits further demineralization by limiting the loss of calcium and phosphate ions. Additionally, SDF promotes the formation of calcium fluoride, which dissolves in the saliva releasing fluoride ions. The alkalinity of SDF creates an ideal environment for ion exchange, facilitating the substitution of the hydroxyl ion in hydroxyapatite with fluoride to form acid-resistant fluorapatite [40]. SDF inhibits matrix metalloproteinases and cysteine cathepsins, limiting the degradation of the dentinal collagen [41,42,43]. The alkalinity of SDF has been shown to enhance the deposition of mineral crystals on exposed collagen in demineralized dentin. It has been postulated that SDF promotes both inter-fibrillar and intra-fibrillar remineralization resulting in the increased microhardness of dentinal lesions [44]. Lastly, SDF demonstrates a potent antimicrobial effect and reduces the formation of a cariogenic biofilm [45,46]. 

### 4.1. Caries-Preventive and Caries-Arresting Efficacy of SDF 

Meta-analysis by Chibinski et al. evaluating the efficacy of SDF in primary teeth, found that the 12-month caries arrest rate with SDF application was 66% more effective compared to other active treatments (ART and FV) and 154% more effective compared to placebo/no treatments. Overall, SDF treatment was found to be 89% more effective than alternate/no treatment in arresting carious lesions [47]. Subgroup analyses have reported that the caries-arresting efficacy of SDF is higher in anterior teeth relative to posterior teeth [48,49,50,51,52]. Baseline oral hygiene is important in determining the caries arrest rate; larger lesions in children with visible plaque were less likely to become arrested, particularly if SDF was applied annually [51,52]. Biannual application of SDF could increase its caries-arresting efficacy in children with poor oral hygiene [49].

Most clinical trials evaluating the efficacy of SDF in primary dentition have been conducted on school children. Mabangkhru et al. assessed the efficacy of SDF vs. 5% sodium fluoride varnish (NaF) in arresting lesions in severe early childhood caries (SECC) in children aged 1–3 years. It was found that use of SDF was two times more likely to result in caries arrest relative to NaF. Decay in anterior teeth was more likely to become arrested than posterior teeth, and occlusal lesions were less likely to become arrested relative to buccal/lingual lesions. Dietary habits governed caries arrest. Children who were weaned from milk feeding and did not snack more than three times a day were more likely to develop inactivated lesions. The authors concluded that SDF can be used to effectively manage SECC [53]. 

In addition to caries arrest, SDF application has a caries-preventive effect and de-creases the incidence of new lesions. The treatment of four primary teeth was found to prevent one new lesion in primary teeth [54]. Meta-analysis comparing the caries-preventive effect of SDF and placebo/no treatment in primary teeth over 24 months or more found the prevented fraction (PF) of SDF to be 77%. A similar Cochrane review for FV found the PF to be 37%. Compared to quarterly application of FV, the annual application of SDF in primary teeth was found to be more effective in preventing new lesions (PF = 54%, after 24 months) [55]. Although these findings suggest that SDF has a superior caries-preventing ability relative to FV, these data must be interpreted cautiously. Trials evaluating the preventing effect of a treatment modality (e.g., FV) usually report new lesions that develop in the entire dentition. However, some SDF trials selectively reported new lesions (Llodra et al. only reported new lesions in posterior teeth, Chu et al. only reported new lesions in anterior teeth). These differences in reporting make direct comparisons of treatment efficacy problematic [56]. A single clinical trial directly comparing GIC to SDF found that SDF performed better than GIC in primary dentition over 12 months, and this difference was statistically significant [57]. To evaluate the caries-preventive effect, long-term trials are needed. 

Few clinical trials have evaluated the caries-arresting and preventing effect of SDF in permanent teeth. Llodra et al. reported that 77% of active carious lesions became inactive in both primary and permanent first molars [33]. Braga et al. compared the decay arresting effect of SDF, toothbrushing and GIC application on incipient lesions in permanent first molars and reported that although SDF was more effective than other methods over the first 6 months, they were equally effective over a 30-month period [58]. A review by Rosenblantt et al. evaluating the caries-preventive effect of SDF concluded that the PF was >60% for permanent teeth [59]. However, Liu et al. found sealants, semi-annual FV and annual SDF, to be equally effective in preventing pit and fissure caries over 24 months [60], whereas Monse et al. found GIC sealants to be more effective than a single SDF application after 18 months in dentinal lesions [61]. Strong evidence to reach a conclusion regarding the caries-arresting and preventing effect of SDF in permanent teeth is lacking [56].

SDF has been used to prevent and arrest root caries in the elderly. Over a 24–36-month period, the PF for SDF was 24% and 71%, respectively. For caries progression, the PF was found to 725% greater than a placebo in a 24-month period and 100% greater in a 30-month period [62].

A retrospective study assessing the 12-month survival outcomes of SDF application amongst patients at a community clinic found that the survival rate of SDF application alone was 76%, whereas SDF with sedative filling and same-day restorations had survival rates of 50% and 84% respectively. SDF with a sedative filling failed at 2.5 times the rate of SDF alone and the lower survival rate was thought to be due to the interim nature of the sedative filling. In primary teeth, SDF alone had highest survival rates in cuspids (83–86%) and lowest rates in lower molars (71%). In permanent teeth, the survival rates of anterior teeth (50%–70%) were lower than posterior teeth (75–82%). Overall, the survival rates of primary (74%) and permanent (78%) were comparable. Survival rates of SDF alone in children under 6 years (69%) and adults over 41 (68–72%) was lower than patients aged 6–40 years (77%–84%). Based on caries risk assessment, the survival rates were found to be 81%, 76% and 75% respectively in low, moderate and high-risk patients. The findings of this population-level, real-world study suggests that SDF application is effective in caries arrest. Since the data for the study was drawn from dental claims, the diagnosis for SDF application (e.g., hypersensitivity vs decay, cavitated vs non-cavitated lesions) could not be ascertained and therefore data only represents overall survival rates of SDF application and must be interpreted cautiously [63].

### 4.2. Limitations of SDF 

Owing to the efficacy and rising popularity of SDF in the pediatric population, it has been rapidly incorporated into both the predoctoral and graduate level curriculum and clinical practice. Additionally, a new code (D1354) has been added by the ADA Code Commission to reimburse providers for using SDF as an interim caries-arresting medicament [26]. 

A 2016 survey amongst U.S. pediatric dentistry program directors evaluating the use of SDF in academic settings showed that 89.2% agreed with the use of SDF in high caries risk patients and only 9.5% disagreed with its use in primary teeth [26]. One of the major advantages of SDF is its ease of application and, as such, pre-cooperative patients, patients needing advanced behavior management techniques and those with limited access to dental care were deemed as good candidates for SDF treatment [26]. Clinically, SDF application causes a permanent black discoloration of the carious enamel and dentin and concerns about parental acceptance of the treatment due to the staining was reported to be the most common perceived barrier to its use amongst healthcare professionals [26,64] (91.8% pediatric dentistry program directors and 56% registered dental hygienists). 

Studies investigating parental opinions and concerns about the acceptability of SDF treatment in different settings have reported contradictory results, mainly due to differences in parental esthetic expectations for their child’s dental care and cultural differences regarding the value of esthetics in primary dentition [65,66]. While Chu et al. reported that all parents were satisfied with the esthetic appearance following SDF treatment and only 7% mentioned the staining, Alshammari et al. reported that all parents refused (‘strongly refused’ or ‘refused’) SDF treatment and only 3.2% remained neutral in their disposition [36,67]. Awareness regarding the availability of esthetic treatment options at hospitals or private institutions contributed to the overwhelming rejection of SDF treatment among these parents [67]. 

In addition to the influence of cultural differences in esthetic expectations, parental acceptance was found to vary with tooth location, level of parental education, income and the need for advanced behavior management techniques [65]. It was observed that although parents found SDF staining acceptable considering the ease of application and child comfort [68,69], they were less inclined to accept the use of SDF in anterior teeth (27%–36%) relative to posterior teeth (54%–69%) [65,66,70] due to esthetic concerns. Acceptance increased among low income groups with public dental insurance compared to groups with private insurance who had more treatment options [65,71]. Moreover, acceptance improved from 54%–62% to 70%–82% among parents with young, pre-cooperative children who had behavior barriers and required treatment to be completed under sedation or general anesthesia. Parents were found to be more willing to accept SDF treatment if it would avoid pain and infection in children and obviate the need for advanced behavior management techniques [65,70,72,73]. Since parents are sensitive to the risks of GA and the associated cognitive defects observed in younger children undergoing prolonged & frequent GA, they were found to be more accepting of treatment alternatives that may prevent or delay the need for GA [66]. Furthermore, parents with higher education were more likely to accept SDF use when considering it as an alternative to treatment under sedation or GA and this difference was attributed to the increased awareness amongst educated parents regarding the additional GA risks [65,66]. This trend of increased SDF acceptance among low income groups and vulnerable populations may cause significant treatment inequities where marginalized groups are more likely to receive treatment with unesthetic outcomes while affluent groups with access to dental care receive conventional esthetic restorations [26].

Although there are limited trials evaluating the effectiveness of SDF in arresting carious lesions in permanent teeth, the ADA has provided conditional recommendation for its use as nonrestorative treatment in permanent dentition, based on indirect evidence of its effectiveness in primary teeth [27]. Nonetheless, 28.3% of the surveyed U.S. pediatric dentistry program directors in 2016 disagreed with the use of SDF in permanent teeth since SDF is a new, unfamiliar material and research regarding its effect in permanent teeth is lacking [26]. Parental acceptance of SDF for permanent dentition was also found to be lower than primary dentition [66,70]. Parents were more willing to accept compromised esthetic outcomes for primary teeth and preferred non-invasive treatment modalities such as SDF over the use of sedation or GA because primary teeth eventually exfoliate. However, they were found to be hesitant to accept unesthetic treatment for permanent teeth, even in settings with limited access to dental care [70,73]. Hu et al. reported that parental esthetic expectations for older children with autism spectrum disorder were similar to those of parents of neurotypical children and parents in both groups were less likely to opt for SDF treatment as an alternative to GA [66]. Thus, parental acceptance of SDF as a caries arrest tool in older children with permanent teeth is limited. 

## 5. Resin-Based Fissure Sealants 

**Figure 1:**
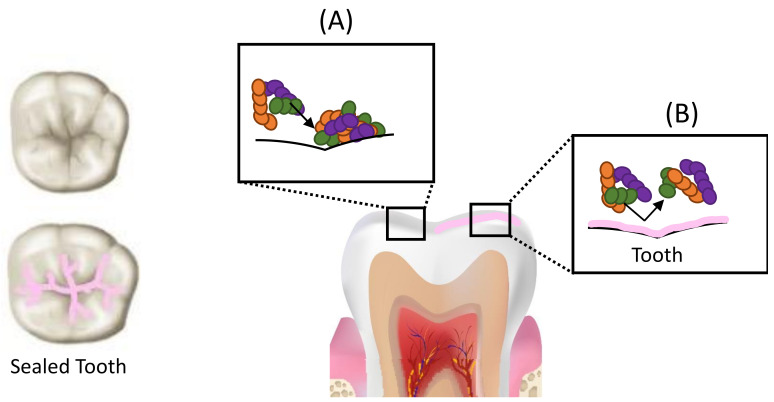
Schematic representation of caries prevention by sealant application. (**A**) Deep pits and fissures on occlusal surfaces of posterior teeth act as retentive sites that facilitate bacterial colonization. (**B**) Sealant application forms a mechanical barrier on the tooth surface and renders it more amenable to cleansing, making it less susceptible to bacterial colonization and preventing dental caries.

Methacrylate-based resin sealants have been used to prevent dental caries since the 1960s. Dental sealants form a mechanical barrier between the enamel and the pathogenic biofilm (Figure 1) and have been shown to be more effective in preventing dental caries in permanent molars relative to unsealed teeth [74].

The relative risk reduction in the development of new carious lesions due to sealants was found to be 87% after 12 months and 60% after 48–54 months [75]. A 10-year study found that only 5.7% of sealed first molars developed caries or needed restorative interventions [76]. The most important criterion determining the success of the caries -preventing effect of sealants long term, was the rate of sealant retention [74,75]. A meta-analysis assessing the longevity of sealants found the retention-rate of light-polymerizing sealants to be 68.4%, 83.1% and 57.8% after 2, 3 and 5 years of follow-up respectively [77]. Complete or partial loss of sealants may provide an avenue for bacterial ingress and furnish a caries-susceptible site; therefore, lost sealants need to be replaced [78]. These findings indicate that the caries-preventing effect of sealants could decrease over time due to sealant loss and underscore the need for regular follow-up visits after sealant application. 

**Figure 2:**
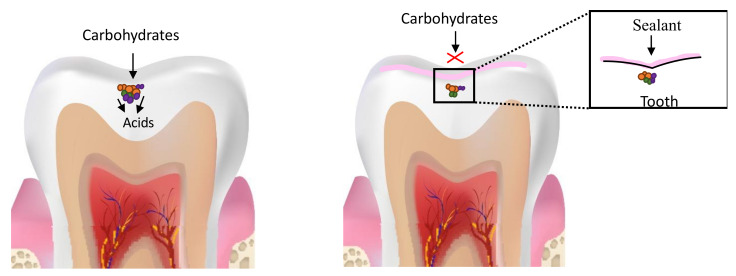
Schematic representation of caries arrest by sealant application. Sealant application deprives the biofilm within the carious lesion of nutrient supply, resulting in a reduction in the number of viable bacteria within the lesion, leading to the formation of a less virulent and less diverse biofilm, slowing/arresting caries progression.

More recently, due to the paradigm shift in dentistry towards less invasive procedures, it has been recommended that the use of sealants be extended to include teeth with carious pits and fissures for the arrest of dental decay [79,80]. The evidence for effectiveness of sealing carious fissures has been documented since the 1970s when it was found that sealing bacteria under restorations results in a reduction in the number of viable bacteria [81]. Moreover, at least a 100-fold reduction in *Streptococcus mutans* and lactobacilli number was observed in sealed lesions [82,83]. Since sealants can form a hermetic seal, the occlusal carious lesion can be isolated from the oral environment, depriving the biofilm of nutrient supply, resulting not only in fewer bacteria but also a less virulent and less diverse biofilm [2,4,83]. The biofilm activity is consequently reduced or altered, slowing caries progression [74,84] (Figure 2).

Clinical trials investigating the effect of sealing occlusal carious lesions of varying depths (non-cavitated and cavitated lesion extending from enamel to half/middle third of dentin) for 24–44 months have shown that sealing is an effective strategy for arresting carious lesions, especially when adequate isolation of the tooth can be achieved [85,86,87], yet, a threshold for when the cavity is considered “too deep” to be sealed successfully remains to be clearly determined [84]. The main concern in sealing “very deep” carious lesions is that although sealant may prevent ingress of bacteria from the external environment, they are unable to fully penetrate the lesion and arrest internal carious activity. Unlike the saccharolytic bacteria associated with enamel lesions that depend on the nutrient supply from the oral environment, the bacteria found in deep dentinal caries are predominantly proteolytic and can degrade the organic component of the dentin to furnish nutrients [88,89]. Therefore, the bacteria in the deeper portions of dentinal lesions can continue to remain viable, even if they are cut off from the external oral environment by sealant application, leading to decay progression. Moreover, progression of dental caries into the dentin causes the undermining of enamel and the compromised mechanical properties of the sealants precludes them from protecting the undermined enamel from breakdown under functional loads [84].

Meta-analysis evaluating the caries progression rate in sealed and unsealed carious (cavitated and non-cavitated) lesions have found the progression rates in sealed lesions (5%) to be significantly lower than unsealed lesions (16.1%). Sealing reduced the likelihood of caries progression by 70% and consistently outperformed fluoride varnishes and no treatment in arresting the progression of dental caries [90]. A 10-year study assessing the effectiveness of bonded and sealed composite restorations placed directly over frankly cavitated lesions extending halfway into the dentin (i.e., material was placed without removal of carious dentin) found that the sealed restorations arrested caries progress and exhibited superior clinical outcomes and longevity compared to unsealed amalgam restorations where caries was completely excavated prior to restoration placement [91]. Another study found no significant difference in the caries progression rates in sealed lesions and teeth restored with conventional composites over a 2–3-year period. Continued caries progression was observed under 10% of the sealed lesions whereas 88% of the sealed lesions were arrested (no decay progression was observed under composite restorations and 14% of the sealants were replaced/repaired) [85]. Thus, although sealing lowers the rate of caries progression and renders the need for immediate invasive restorative treatment unnecessary, sealants may be lost and need replacement. Arrested lesions/portions of the inactive lesion may become active and continue to progress, necessitating surgical intervention. These findings highlight the need for persistent, vigilant monitoring of sealed carious lesions.

Despite compelling evidence to support the use of sealants to arrest pits and fissures caries, their use is contended due to concerns about sealant retention, insufficient depth of sealant penetration, incomplete sealing of carious fissures which may lead to undetected decay progression [92] and gaps at the sealant-tooth interface due to polymerization shrinkage which may facilitate plaque accumulation and marginal leakage [93,94]. Adapting sealants to carious fissures is more challenging relative to sound fissures due to the surrounding demineralized enamel and dentin which compromises sealant adhesion [95]. Moreover, carious fissures are difficult to access/clean and conducive to biofilm accumulation. Incompletely cleaned fissures and presence of biofilm in its deeper parts not only leads to unfavorable marginal adaptation [96] but also precludes complete penetration of sealants, leaving unfilled spaces that further facilitates biofilm growth. Inadequately adapted sealants that exhibit increased microleakage and incomplete fissural filling may allow undetected caries progression [93]. Finally, surface degradation of sealants and deterioration of the matrix-filler interface following wear could contribute to sealant loss, rendering the tooth susceptible to caries [97].

### Development of Antimicrobial Sealants

The main shortcomings of resin-based sealants are (1) potential for microleakage due to polymerization shrinkage [98], (2) increased biofilm accumulation around resin-based materials relative to other materials [99], (3) Incomplete filling of carious fissures and poor marginal adaptation [96]. All these limitations facilitate biofilm formation and could contribute to the development of secondary caries. Additionally, clinicians occasionally inadvertently/intentionally seal incipient lesions. Although sealants can reduce the number of viable bacteria, they do not eliminate all cariogenic bacteria. Therefore, addition of antibacterial agents could potentiate the caries-preventing and caries-arresting efficacy of sealants [98].

Both releasing systems and contact-killing mechanisms have been employed to improve the antibacterial properties of resin-based sealants. Releasing systems with fluoride have been developed due to the known anti-caries effect of fluoride including a reduction in the metabolic activity of microorganism, the inhibition of tooth demineralization by inducing fluorapatite formation and the facilitation of remineralization [100]. However, currently, there is insufficient evidence to suggest that fluoridated sealants are superior at preventing caries relative to non-fluoridated sealants [101]. Several studies have found that the frequency of development of new lesions in teeth sealed with fluoridated sealants was similar to that of traditional sealants [102,103]. Moreover, the rate of retention of fluoridated sealants was found to be comparable [104] or lower [105,106] than that of traditional sealants. It has also been found that maintaining the release kinetics of fluoride for sustained antimicrobial effects using releasing systems is a challenge, since these systems usually exhibit a high level of initial release which tapers over time, reducing antimicrobial activity [100,107]. The dissolution of soluble fluoride salts has also been implicated in the diminished mechanical properties of these sealants [107].

Chlorhexidine (CHX) has been incorporated in releasing systems, however, similar to fluoride, the mechanical properties of CHX-releasing sealants reduced over time and the porosities in the material structure due to CHX release made it susceptible to staining, poor wear resistance and biofilm accumulation [108]. In a 6-month in vitro study, Shafiei et al. found that the application of CHX increased microleakage in sealed teeth, increasing its susceptibility to secondary caries [109].

**Figure 3:**
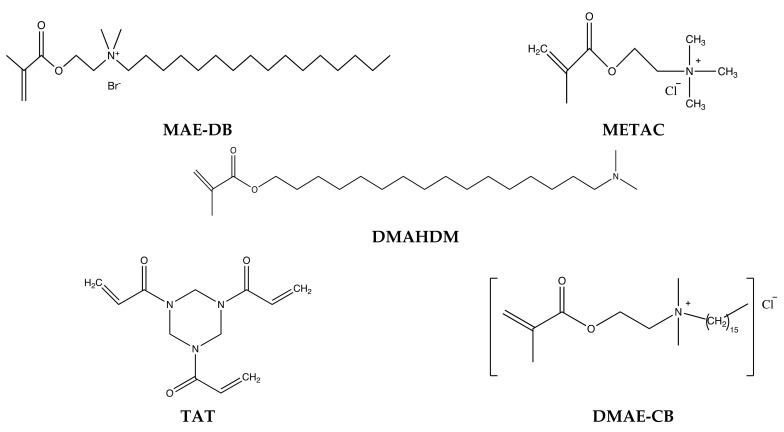
Chemical structures of Quaternary Ammonium Compounds (QACs). 2-methacryloxylethyl dodecyl methyl ammonium bromide (MAE-DB), 2-methacryloyloxyethyl trimethylammonium chloride (METAC), dimethylamino- hexadecyl methacrylate (DMAHDM), 1,3,5-triacryloyl hexahydro-1,3,5-triazine (TAT), methacryloxylethyl cetyl dimethyl ammonium chloride (DMAE-CB).

Contact-killing mechanisms utilizing quaternary ammonium compounds (QACs) have been gaining popularity in recent times since they can be copolymerized within the resin matrix without altering the mechanical and physicochemical properties of sealants [110]. Several QACs including 2-methacryloxylethyl dodecyl methyl ammonium bromide (MAE-DB) [111], 2-methacryloyloxyethyl trimethylammonium chloride (METAC) [112], methacryloxylethyl cetyl dimethyl ammonium chloride (DMAE-CB) [98], dimethylamino- hexadecyl methacrylate (DMAHDM) [113,114] and 1,3,5-triacryloyl hexahydro-1,3,5-triazine (TAT) [115] have been incorporated in sealants to enhance their antimicrobial properties (Figure 3). In vitro evaluations of experimental sealants with QACs have shown encouraging results indicating that addition of QACs did not negatively impact sealant properties such as degree of conversion, infiltrating properties, ultimate tensile strength and micro-shear bond strength [110]. These studies also suggest that since the QACs are covalently bonded to the resins, they do not leach out and therefore their antimicrobial activity should not attenuate over time. However, it is well known that the activity of QACs is reduced by the presence of organic matter such as dead cells and coatings formed by adsorption of salivary proteins [108]. Degradation of the sealant resin in the oral environment over time may also cause the QACs to leach from the material, raising cytotoxicity concerns. Hence, long-term studies analyzing the properties of QAC containing sealants are needed.

## 6. Resin Infiltration for the Management of Carious Lesions

### 6.1. Analysis of Considerations for Use in Non-Cavitated and Cavitated Proximal Lesions

**Figure 4:**
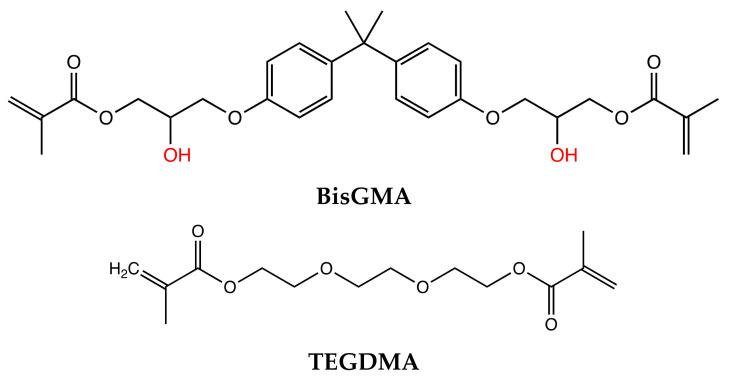
Chemical Structure of resin monomers. Bisphenol A-glycidyl methacrylate (BisGMA) and Triethylene glycol dimethacrylate (TEGDMA).

Resin infiltration has been developed as a minimally-invasive intervention to bridge the gap between the “wait and watch” and “drill and fill” approach to the treatment of interproximal caries [116]. Resin infiltrants (RI) are low-viscosity triethylene glycol dimethacrylate (TEGDMA) based resins that exhibit high caries permeating ability and a high degree of conversion (DC) [117] (Figure 4).

**Figure 5:**
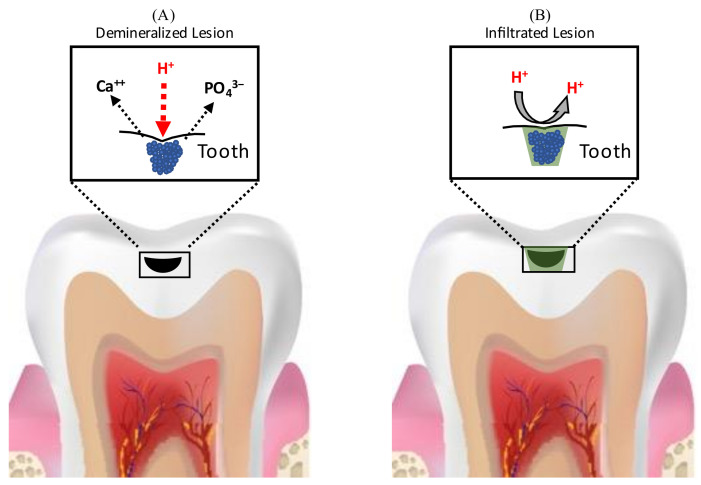
Schematic representation of caries-arresting effect of resin infiltration. (**A**) Demineralization of enamel due to acids, resulting in the formation of enamel porosities (**B**) Resin infiltrants penetrate the demineralized enamel lesions and occlude the inter-crystalline spaces after polymerization, resulting in the formation of a polymer framework that acts as a barrier for hydrogen ions, inhibiting further demineralization and arresting the progress of caries.

RIs penetrate the demineralized enamel lesions and occlude the inter-crystalline spaces after polymerization, resulting in the formation of a polymer framework that micromechanically interlocks the remaining enamel prisms and acts as a barrier for hydrogen ions, inhibiting further demineralization and arresting the progress of caries [117,118,119,120] (Figure 5).

The ability of the Resin infiltrant (RI) to inhibit caries progression depends on its penetration coefficient (PC) (the rate at which a liquid penetrates a capillary or porous bed; PC is directly related to surface tension and inversely related to the contact angle and liquid viscosity) and penetration depth [121]. Penetration in enamel lesions may depend mainly on the viscosity of the RIs, however, penetration in dentinal lesions may depend on both viscosity and hydrophilicity of the material. Research investigating the impact of hydrophilicity of RIs on their ability to infiltrate dentinal lesions is lacking. Attempts have been made to enhance the infiltration ability of methacrylate-based resins by altering their monomer chemistry and adding solvents. In terms of chemistry, owing to its low viscosity, resins with high triethylene glycol dimethacrylate (TEGDMA) concentrations have been found to have superior caries-infiltrating ability and show better caries-inhibition relative to those with high bisphenol A-glycidyl methacrylate (BisGMA) [121]. Alcohol addition is also an effective way to reduce resin viscosity, however, it has been shown to promote the formation of microgels at the sites of polymerization initiation, which reduces the mobility of the generated free radicals, decreasing polymerization [122,123]. Decreased polymerization reduces the mechanical properties of the RIs and limits their ability to inhibit further demineralization of the carious lesion [124], therefore solvent addition to RI is not recommended.

Meta-analysis assessing the effectiveness of resin infiltration in arresting the progress of non-cavitated proximal lesions have found them to be highly efficacious in permanent teeth when the lesions extend into the enamel and outer third of dentin [125,126,127,128,129,130]. For permanent teeth, the risk of caries progression was shown to be significantly lower in infiltrated lesions (4–14%) relative to control (42–48%) over three years [131,132] and Paris et al. found the seven-year rate of progression to be 9% for infiltrated lesions vs 45% for control lesions [133]. Sub-group analysis of RI efficacy in arresting lesions of varying depths showed that while RI was successful in arresting enamel lesions, for dentinal lesions the caries progression rate was not significantly different from control group [128]. The differential treatment efficacy was attributed to differences in the histology and pathology of enamel and dentin caries and is discussed further in a later section.

For primary teeth, early meta-analysis found the data regarding RI efficacy inconclusive, due to the heterogeneity of study designs and settings [125,126,127]. A meta-analysis by Chen et al. in 2021 concluded that although more clinical studies were needed, findings from current studies were encouraging and resin infiltration is efficacious in arresting decay progress in primary teeth for 12–24 months [130]. Based on these studies, the therapeutic effect of RI was found to range from 21% [134,135] to 38% [136].

The success of RIs in arresting the progress of non-cavitated proximal carious lesions has instigated further research exploring the possibility of expanding their use in the treatment of cavitated lesions. For the successful treatment of cavitated lesions using resin infiltration, the RI must be able to not only infiltrate the demineralized portion of the lesion, but also fill the cavitated portion of the lesion. Studies analyzing the effectiveness of RIs in deep and micro-cavitated proximal lesions (International Caries Detection and Assessment System (ICDAS) 3,4,5) found that while RIs can adequately infiltrate demineralized porous enamel in all carious lesions regardless of depth, they could not fill the cavitated portions of larger lesions [137]. The inadequate filling of the cavitated lesions was attributed to three mechanisms: First, porous demineralized enamel has strong capillary forces that drives the perfusion of RI into the pores, while large cavities have weak capillary forces and therefore do not induce similar filling. Second, the cleaning procedure prior to light curing may partially remove some RI from the cavitated lesions. Lastly, penetration of the RI into the cavitated lesions may be impeded by the surface tension of the air bubbles trapped within This inadequate filling leads to the formation of a thin and in-homogenous resin layer in deeper lesions (ICDAS 4&5) compared to ICDAS 2&3 lesions. The caries-inhibiting ability of the resin layer is correlated to the thickness of the resin layer and therefore the thin layer is less efficient at inhibiting demineralization in deeper lesions. Moreover, since incomplete filling of the cavities facilitates biofilm accumulation, it further reduces the caries-inhibiting efficacy of RI in deeper lesions [137].

To enhance the caries-arresting effect of resin infiltration in deep cavitated lesions, (1) its ability to fill cavities must be improved without diminishing its infiltrating capabilities [138,139]. One strategy to improve the filling ability of the RI is to modify its mechanical properties with the addition of fillers such that its viscosity and mechanical properties are optimized while its penetration capability is maintained [138,139]. (2) it may be endowed with antimicrobial properties to increase its ability to eliminate the bacteria within the incompletely filled cavitated lesions and prevent bacterial re-infection.

### 6.2. Micro-Filled Infiltrant Resins (MFIR)

RIs have poor mechanical properties such as low mechanical strength, high polymerization shrinkage and low wear resistance. Due to their low viscosity, they have inadequate filling abilities.

To address these issues, micro-filled infiltrant resins (MFIRs) were developed by adding fillers (glass and organic) to RIs since the addition of fillers has been shown to improve the resin mechanical properties such as flexure strength and modulus of elasticity. Filler also reduces polymerization shrinkage and water sorption [140,141]. Ideally, MFIRs should have the infiltrating properties similar to RIs and filling properties comparable to flowable composites.

#### 6.2.1. Effect of Fillers on RI Properties

When fillers are added to resins, the properties of the resultant resin are influenced by several factors such as the filler shape, size, concentration and interactions between filler particles and resin matrix [142]. When the filler content is low, the inter-particle interaction is weak and as the number of particles increases, they become closely packed and exert stronger inter-particle interactions, increasing resin viscosity [142]. Regarding particle size alone, the addition of very small sized filler particles increases the viscosity of the material more compared to the addition of large filler particles due to the increased surface area and interaction between the filler particle and matrix. The incorporation of larger filler particles, on the other hand, results in the formation of in-homogenous resins with impaired enamel wetting ability, resulting in compromised penetrating properties [139,143].

#### 6.2.2. Factors Affecting the Movement of RI from Micro-Filled Infiltrant Resin (MFIR)

When the MFIR is applied to a carious lesion, there are two competing phenomena affecting the flow of the infiltrant resin: capillary forces drive the RI from the MFIR to the pores in the demineralized lesion, while the interfacial interaction between the filler particles and the RI in the MFIR restricts the RI’s movement. As the interfacial surface area increases, conversely related to the size of the filler, the amount of RI needed to coat the filler particles increases, reducing the amount of free resin available to infiltrate the carious lesion [138,139,143].

Studies evaluating the effectiveness of MFIR modified with pre-polymerized methacrylate-based fillers (42 μm) in deeper lesions (ICDAS 3&5) found no significant difference in the penetration ability of RI and MFIR. Additionally, the filling ability of MFIR was higher (100% for both groups) than RI (25% for ICDAS 3 and 38% for ICDAS 5). MFIRs had infiltrating properties comparable to RI and filling properties comparable to the flowable composite. This was observed because when MFIR is applied to a carious lesion, the resin infiltrates the carious lesion while the filler is left embedded in the remaining surface monomer, which acts in the same way as flowable resin and fills the cavities [138,139]. These findings suggest that MFIRs may present a promising approach to inhibit caries progression in deep and cavitated proximal lesions.

### 6.3. RIs for Arresting Occlusal Carious Lesions

In expanding the scope of their use, RIs’ application to arrest occlusal carious lesions has also been investigated. However, due to the poor mechanical properties the use of RIs alone is not recommended for the arrest of occlusal carious lesions [139,144,145]. In vitro studies have advocated the use of micro-filled infiltrant resins (MFIR) or RIs in combination with conventional sealants/flowable composites applied overtop of the infiltrated tooth structure for this purpose [139,145]. These studies have demonstrated that the use of MFIR or RIs with conventional sealants/flowable composites for the arrest of occlusal carious lesions offer several advantages over conventional sealants: First, the diffusion barrier is shifted from the enamel surface to the body of the carious lesion and therefore even if the sealant is lost or does not remain completely intact, the infiltration of the body of the lesion will remain and therefore could prevent the progression of the carious lesion. Second, the ability of RIs to penetrate into carious fissures is superior to that of conventional sealants due to higher penetration coefficients and more intense surface erosion with 15% hydrochloric acid for RIs which facilitates deeper infiltration [139,146]. (Although HCl effectively removes the surface layer of the carious lesion, it must be used cautiously in the oral environment since it may cause accidental injuries to the soft tissue). Third, use of RIs with flowable composites has been shown to reduce immediate microleakage more effectively compared to conventional sealants [147]. Thus, the use of RIs in combination with sealants can mitigate some of the challenges of conventional sealing in carious occlusal fissures including retention, poor penetration and microleakage. Hence, this may be an effective minimally invasive approach to treat carious fissures. The addition of antimicrobial agents to RIs can aid in eliminating the bacteria trapped within the deeper portions of the lesions and could further enhance the caries-arresting efficacy of this treatment modality.

Clinical trials evaluating the effectiveness of RIs in arresting occlusal carious lesions have found that sealing and infiltrating occlusal lesions in combination with fluoride varnish application was highly efficacious in arresting carious lesions relative to varnish application alone in primary dentition over a 2–3 year period [148]. Anauate-Netto et. al., demonstrated that the caries arresting efficacy of both infiltrating and sealing non-cavitated occlusal lesions was comparable over a 3 year period in permanent dentition [149]. In contrast to these findings, Elkwatehy et al. found that in sound and non-cavitated permanent molars, sealing alone and a combination of infiltrating and sealing occlusal lesions was more effective in preventing and arresting the progress of carious lesions whereas the use of RI alone was not effective [150]. The differences in these studies could be attributed to variations in the depth of selected lesions. While the former study included deeper lesions (most ICDAS 2), the latter selected teeth with ICDAS 0,1,2 lesions. It has been shown that sound fissures (ICDAS 0) and incipient lesions (ICDAS 1) may not benefit from infiltration compared to conventional sealants whereas deeper lesions (ICDAS 2) demonstrate higher penetration of RI and consequently superior caries-inhibiting effect compared to sealants alone.

### 6.4. Limitations of Current Resin Infiltrants

Although RIs present an effective treatment modality, their use is associated with a few short comings.

#### 6.4.1. Incomplete Lesion Resin Infiltration/Penetration

Despite effective penetration of carious lesions by RIs, deeper enamel lesions have been observed to demonstrate in-homogenous and incomplete infiltration compared to the total depth of the lesion [119,151]. Only 60 % of the enamel pore volume was found to be sealed by RIs in advanced lesions [119,152] and the microhardness of the restored lesion was not comparable to that of sound enamel [153], making the restored lesions susceptible to new cariogenic challenges [153,154].

Analysis of RI efficacy on lesions of varying depth showed that while RI was 100% successful in arresting lesions in inner enamel, for lesions extending to outer dentin the success rate reduced to 64% [131]. In a sub-group analysis, it was found that when lesions extended into the dentin, there were no differences in the caries progress rates for RI group and control groups [128]. The resin penetration of RI in carious dentin (82%) is lower than carious enamel (99.1%) and the inability of RIs to fully permeate dentinal lesions results in poor therapeutic efficacy [128]. The differential treatment efficacy of RI for enamel and dentinal lesions is due to differences in the composition, histology and caries pathology of these tissues. Enamel is a highly mineralized tissue consisting mainly of hydroxyapatite crystals. Enamel caries occurs due to the production of organic acids by cariogenic bacteria that causes demineralization, resulting in enlargement of enamel pores. Bacterial invasion in enamel is always preceded by the enlargement of pores and when resin infiltration is used, it occludes the enlarged pores and creates a diffusion barrier, preventing the passage of nutrients from the oral cavity toward the cariogenic bacteria trapped within the lesion, isolating the mineral tissues from cariogenic acids, as well as keeping the dissolved minerals within the affected tissues, increasing the likelihood of remineralization once the bacteria are deprived of nutrients [92,155,156,157]. As a result, this resin layer increases the demineralization resistance and microhardness of the enamel and consequently inhibits the progression of carious lesions [153,158]. Dentin on the other hand has a higher proportion of water and organic matter (40%). Additionally, dentinal tubules facilitate bacterial invasion, therefore, dentinal caries is more complex and progresses at a faster rate. Along with dissolution of the inorganic matter by acids, dentin caries involves the degradation of organic matter by bacterial proteolytic and hydrolytic enzymes [88,89]. These degradation products could decrease the dentin wettability. Also, the demineralized dentinal tubules adjacent to the infected enamel may provide enlarged pathways for the passage of dentinal fluid into the proximal carious lesion and preclude complete drying, rendering complete infiltration with RIs difficult in dentinal lesions [159]. Finally, although the RI barrier could isolate the dentinal lesion from the bacteria in the external environment, its effect on the bacteria within the lesion remains to be investigated [128].

#### 6.4.2. Surface Roughness

Subsurface demineralization creates porosities between enamel rods leading to surface roughness. Resin infiltration does not restore the roughness of the lesion to that of sound enamel and is not amenable to polishing [160,161]. Studies have found that the roughness of restored surface continues to remain higher than that of sound enamel and therefore may facilitate biofilm accumulation [153,162,163,164]. Surface texture evaluation following treatment with adhesives and RIs showed that adhesives formed a more homogenous layer relative to RI due to their superficial penetration [165]. Results of an in vitro study comparing the effect of fluoride varnish, nano-hydroxy apatite paste and resin infiltration on *S. mutans* adhesion to artificial enamel lesions found that resin infiltration was associated with highest levels of bacterial adhesion amongst the three groups [166].

#### 6.4.3. Polymerization Shrinkage and Microleakage

Owing to the low molecular weight monomer TEGDMA, which is the main constituent of RIs, they exhibit high polymerization shrinkage and polymerization stresses, leading to microleakage, which could decrease their caries-arresting efficacy and increase susceptibility to secondary caries [138].

#### 6.4.4. Leached Monomer Cytoxicity

It is well established that residual, unpolymerized monomers leach from the surfaces of the restorations during photopolymerization and continue to leach after the restorations harden [167]. Samuelsen et al. demonstrated that 24 h exposure of very low concentrations (0.5 mM) of TEGDMA results in cell death [168]. Furthermore, Batarseh et al. demonstrated that human pulp fibroblasts exposed to TEGDMA (0.25 mM) showed significant increases in pro-apoptotic proteins such as Cytochrome c, Caspase 3 and Bim at 24 h [169]. Although significant concentrations of TEGDMA is eluted from RIs outwards, into the oral cavity, it occurs for the first few minutes and hence 24 h contact time is unlikely [170]. Currently, resin infiltration is only used to restore enamel and dentinal lesions in the outer third of dentin. Therefore, pulp-ward diffusion of TEGDMA is unlikely. However, if resin infiltration is used to restore deep lesions (ICDAS 5), inward diffusion of TEGDMA may occur and result in untoward pulpal outcomes. Subtoxic doses of TEGDMA (0.3mM) have been shown to reduce the expression of mineralization-related genes by 5–20% after 4 h and 50% after 12 h. TEGDMA may inhibit pulp induced mineralization and impair the formation of reparative dentin [171]. Hence, these materials must be used cautiously in deep dentinal lesions.

#### 6.4.5. Methacrylate Resin Degradation

In addition to the diffusion of unreacted monomers, methacrylate resins are susceptible to water sorption, causing monomer hydrolysis and cleaving of ester bonds [172]. An in vitro analysis by Arslan et al. comparing the effects of aging on RI, dental adhesives and fissure sealants by subjecting the restorations to 10,000 cycles of thermocycling (equivalent to 1 year of aging) found that RIs were more susceptible to water sorption relative to dental adhesives and fissure sealants [173]. This may be due to the higher proportion of hydrophilic TEGDMA monomer in RIs and may impact the longevity of resin infiltration treatments in the oral environment. Furthermore, resin infiltrated lesions have been shown to develop micro-cracks because of internal stresses caused by thermal expansion and contraction [174]. These micro-fissures provide an avenue for the ingress of salivary and bacterial enzymes which in turn promote the degradation of TEGDMA [175,176]. The degradation by-product of BisGMA, a commonly found monomer in dental adhesives, is 2,2-bis[4(2,3-hydroxypropoxy)phenyl] propane (bisHPPP) and the degradation by-product of TEGDMA, a commonly found monomer in RI, is Triethylene glycol (TEG) [177]. BisHPP has been shown to slightly inhibit the growth of *S. mutans* while also increasing its virulence by upregulating the genes facilitating bacterial attachment and survival in low-pH environment [178]. Triethylene glycol (TEG), has been shown to stimulate the growth and pathogenicity of *S. mutans* [179,180] and increase the expression of esterases [181]. Esterases produced by cariogenic bacteria further degrade resins [175,176], increase surface roughness and promote further bacterial accumulation [182], setting up a vicious cycle of biofilm accumulation and resin degradation, leading to recurrent caries.

To improve the surface topography of resin infiltrated lesions and protect the restored lesions from the effects of biofilm accumulation, polymerization shrinkage related microleakage, and hydrolysis related degradation in the oral environment, Rai et al. coated the surfaces of resin infiltrated lesions with chlorhexidine (CHX) varnish and found that for deeper lesions (ICDAS 3) over a 9 month period, combined treatment of RI and CHX was more efficacious in inhibiting caries progression compared to RI alone. However, for ICDAS 2 lesions, no difference in efficacy was observed [183]. Meyer-Lueckel et al., demonstrated that superficial penetration correlates to the formation of a more homogenous layer [132]. This may explain why the benefits of varnish application were evident in ICDAS 3 lesions alone which necessitated deeper RI penetration. To study the benefits on resin-degradation and microleakage a long-term study may be needed. Nonetheless this study shows that the caries-arresting efficacy of RIs in deeper lesions can be improved by enhancing their antimicrobial properties. Incorporating antimicrobial agents within the RI may have added benefits since it could additionally eliminate the bacteria trapped within the lesion. Use of antimicrobial releasing systems could increase the concentration of antibacterial agent in the localized micro-environment of the tooth and prevent bacterial accumulation on virgin proximal tooth surface adjacent to the restored teeth, levying a protective effect and contributing to the caries-preventing potential of RIs. However, any secondary protective effect on adjacent healthy tooth is highly dependent on the kinetics of antimicrobial release from the restoration and diffusion through and dilution by saliva.

### 6.5. Improving the Antimicrobial and Anti-Degradative Properties of Resin Infiltrants (RI)

Given the susceptibility of incompletely infiltrated advanced lesions to new cariogenic challenges, reduced ability of RI to arrest dentinal lesions, increased surface roughness of infiltrated lesions, polymerization shrinkage-related microleakage and the time-dependent degradation of TEGDMA, enhancing the antibacterial and anti-degradative properties of the restorative material may be a promising approach for improving its caries-arresting efficacy in deeper lesions and increasing its longevity by limiting its deterioration in the oral environment.

Commercially available RIs (e.g., Icon^®^) have TEGDMA as their main constituent monomer. TEGDMA is hydrophilic and susceptible to water sorption and solubilization by saliva, leading to hydrolytic degradation in the oral environment, which diminishes their mechanical properties and clinical performance over time [172]. Since the behavior of the polymers is influenced by the chemical characteristics of the monomers, several attempts have been made to improve the mechanical and antibacterial properties of RIs by utilizing different monomer blends [123,184,185,186,187].

**Figure 6:**
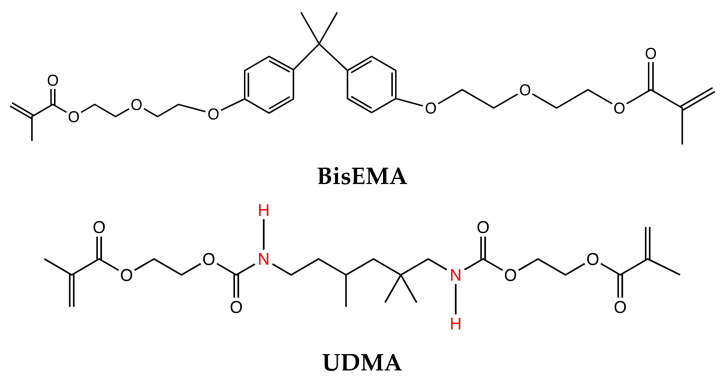
Chemical Structure of resin monomers. Bisphenol A ethoxylate dimethacrylate (BisEMA) and Urethane dimethacrylate (UDMA).

Inagaki et al. studied the effects of adding hydrophobic monomers such as bisphenol A ethoxylate dimethacrylate (BisEMA) and urethane dimethacrylate (UDMA) to TEGDMA along with varying concentrations of Chlorhexidine (CHX 0.1% & 0.2%). The mechanical, anti-degradative and antibacterial properties of experimental RIs were evaluated and compared to Icon^®^ [184,185] (Figure 6). To assess the mechanical properties, the degree of conversion (DC) and microhardness was measured (knoop hardness number (KHN). It was found that the addition of BisEMA and UDMA increased the DC and KHN of the experimental resins relative to Icon^®^ and TEGDMA/UDMA based blends showed the highest KHN values. Moreover, the addition of CHX did not affect DC and KHN at the concentrations tested [185]. The water sorption values of TEGDMA/BisEMA were found to be comparable to Icon^®^ whereas values for TEGDMA/UDMA based blends were significantly higher than Icon^®^ [184]. The solubility of all resin blends was significantly less than Icon^®^ [184] whereas the homogeneity of penetration of all the resin blends (70%–100%) was found to be comparable to Icon^®^ (100%) [123].

These findings suggest that the addition of BisEMA and UDMA to TEGDMA may improve some its mechanical properties while maintaining resin-penetrating properties. Hence, this and other chemical modifications could be explored to further improve the mechanical properties and biostability of RIs.

In the aforementioned studies, the addition of CHX enhanced the immediate antibacterial activity of all resin blends against *S. mutans*, and it was found to be higher than Icon^®^, which demonstrated no antimicrobial activity. However, the rate and duration of release of CHX from the resin blends was not evaluated. Previous studies have shown that releasing systems often demonstrate a short-term, burst release [187], whereas for lasting antimicrobial effects, a sustained release over a prolonged duration is necessary. Therefore, long-term studies evaluating the efficacy of resin blends releasing antimicrobial agents are needed to demonstrate their benefits over time. An immediate antimicrobial activity upon application of the RI may still be useful in killing and disabling cariogenic bacteria trapped under the material during application. However, in the long term, the release of antimicrobial agents could result in porosities within the material, compromising the mechanical properties of the material [108].

**Figure 7:**
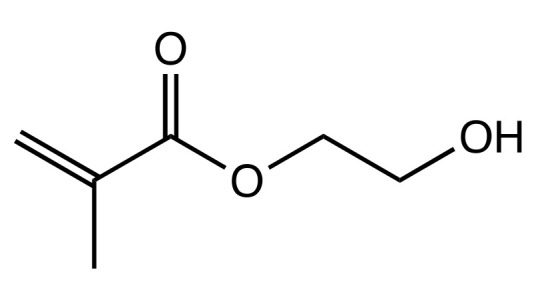
Chemical Structure of resin monomer. Hydroxyethyl methacrylate (HEMA).

Along with releasing systems, contact-killing approaches have also been explored. Marchi et al. sought to enhance the mechanical and antibacterial effect of TEGDMA/BisEMA based RI (HEMA diluent) with the addition of iodonium salt and chitosan [186] (Figure 7). Onium salts were added to improve the mechanical properties of the material since these water-soluble salts have been shown to promote the polymerization of hydrophilic monomers owing to their ionic nature, resulting in a higher conversion of C=C bonds. The formation of a highly cross-linked polymer reduces the free space in its network, making it less susceptible to water sorption and hygroscopic expansion, yielding a polymer more resistant to hydrolytic degradation, even in the presence of hydrophilic monomers like TEGDMA [188]. Chitosan-a polysaccharide- was incorporated due to its antimicrobial effect and its ability to inhibit tooth demineralization [189]. In this study however, the addition of ionic salt did not improve the DC, most likely due to rapid rate of polymerization [186]. Low DC could compromise the mechanical properties of the RI long-term and therefore require further studies.

**Figure 8:**
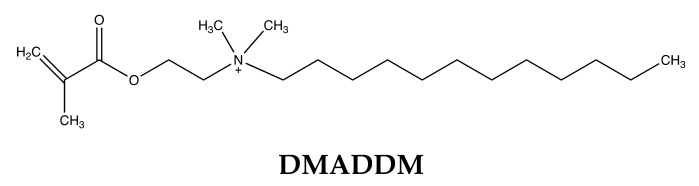
Chemical Structure of quaternary ammonium monomer. Dimethylaminododecyl methacrylate (DMADDM), n = 11.

Yu et al. incorporated dimethylaminododecyl methacrylate (DMADDM), a quaternary ammonium monomer (QAM) by copolymerizing it with the RI, thereby immobilizing it within the resin by the formation of covalent bonds [190] (Figure 8). QAMs have been extensively studied and have been shown to exhibit bactericidal effects and reduced bacterial adhesion. Since they are immobilized within the resin, unlike releasing systems, their antimicrobial activity is not supposed to diminish over time. However, the effects of QAMs requires contact with the surface of the bacterial cell and its effect on the microorganisms in the bulk of a formed biofilm needs to be investigated [187]. Moreover, their effects are usually bacteriostatic and weaker to those of releasing systems. The efficacy of contact mediated killing is also reduced in the presence of organic coatings formed by the adsorption of salivary proteins on resin surfaces [108]. In this study, the addition of QAMs preserved but did not improve the mechanical properties of the RI making them prone to water sorption and degradation in the oral environment. Since the QAM is linked to the resin backbone, RI degradation may lead to their leaching in the oral environment over time. If this occurs, the antimicrobial effect of the RI may diminish in the long term and it also raises toxicity concerns.

Some studies have added filler particles to improve the mechanical and antimicrobial properties of RIs [159,191]. Metallic nanoparticles (NPs) have gained popularity due to their inherent antimicrobial effects [191,192]. Prior studies have demonstrated the antimicrobial effect of Zinc Oxide (ZnO) [193] and silver (Ag) [194,195] NPs on *Streptococcus mutans* and Lactobacillus. Therefore, ZnO and AgNPs were incorporated in RIs to enhance their antimicrobial properties. Kielbassa et al. studied the effect of filler addition on the infiltrating properties of RI. AgNPs were added to RIs to synthesize modified RIs. Tunnel approach was used for conservative tooth preparation and the cavity was restored using the RI and flowable composite (internally). Additionally, the external surface was infiltrated with RI or modified RI. The study found that none of the lesions were fully infiltrated (regardless of AgNP) and the penetrating ability of RIs was not altered by the addition of AgNPs [159]. Angel Villegas et al. found similar effect with the addition of Zn oxide NPs. When ZnO NPs were suspended in RIs, Zn was found up to depths of 1020 μm from the tooth surface whereas no Zn penetration was observed when phosphate buffer solution was used as a carrier [191]. These studies indicate that filler particles may be used to improve the antimicrobial properties of RIs without negatively impacting their infiltrating properties.

## 7. Chemomechanical Management of Caries

Chemomechanical agents such as sodium hypochlorite-based Carisolv and papain enzyme-based agents such as Papacarie and Brix 3000 have been used for the selective removal of infected dentin in primary and permanent teeth [196,197]. Alpha-1-antitrypsin found in healthy tissues prevents collagen breakdown by proteolytic enzymes. Since infected dentin lacks alpha-1-antitrypsin, the proteolytic enzymes found in chemomechanical agents can degrade the collagen in infected dentin, allowing for the selective removal of denatured collagen [198]. Systematic review and meta-analysis comparing the effectiveness of Papacarie and conventional excavation in primary teeth found that Papacarie was efficacious for selective carious tissue removal and was associated with significantly less pain relative to conventional excavation. However, its decay excavation times were longer [199]. The findings of a recent in vitro investigation comparing the efficiency and efficacy of Papacarie, Brix 3000 and conventional excavation corroborated with these findings and concluded that while all approaches were effective in the removal of infected dentin, use of conventional excavation was more efficient (54 s) relative to Papacarie (110.5 s) and Brix 3000 (85 s) [198] but associated with more pain.

## 8. Atraumatic Restorative Treatment (ART)

Atraumatic Restorative Treatment (ART) was developed as a treatment approach for restoring caries in developing countries where rendering definitive treatment may be difficult due to lack of resources [200]. It involves the removal of infected tooth structure with hand instruments without the use of anesthesia followed by the filling of the cavity with a fluoride-releasing restorative material such as glass ionomer cement (GIC) or resin modified glass ionomer (RMGI) [201]. The fluoride release from the GIC induces the formation of fluorapatite which is more acid-resistant, making the tooth less susceptible to future caries [202,203]. GIC can be recharged and acts as a reservoir of fluoride ions taken up from topical applications [204,205]. More recently, the use of SDF with ART has been propagated [206]. The most common problems associated with ART are marginal gaps, lack of wear resistance and loss of restorations [207,208].

Meta-analysis assessing the survival rates of ART have found that for primary posterior teeth, they were 94.3% (±1.5) for single surface restorations and 65.4% (±3.9) for multi-surface restorations over 2 years. For permanent posterior teeth, the survival rates were 87.1% (±3.2) for single surface and 77% (±9.0) for multi-surface restorations over 3 and 5 years respectively [209].

ART has been used in the treatment of Early Childhood Caries (ECC) since it does not require administration of GA and prompt, early intervention is key in the treatment of ECC. Silva et al. found the 4-year success rate of ART in children (18–36 months) to be 94 %, 87.5% and 82.9% in 1,2- and 4-year follow-up period. Faccin et al. also assessed ART success amongst preschool children (average age 31 months) and found lower yet acceptable rate of 72% over 25–48 months [210]. These findings show that ART is an effective treatment modality for ECC and yields excellent clinical outcomes.

Attempts have been made to improve the efficacy of ART with the addition of antimicrobial agents. To reduce the number of residual bacteria remaining in the cavity after the removal of infected tissue, CHX was added to GIC. An in vitro study found that incorporating CHX in GIC significantly lowered the residual populations of *S. mutans* and Lactobacillus in the cavity over 3 months [211]. In vivo studies showed that while the addition of CHX significantly improved the antibacterial properties of GIC, it increased failures due to marginal defects after 9 months [212]. Further research into improving the antimicrobial properties of ART restorations without negatively impacting their mechanical properties is warranted.

## 9. Conclusions

Several minimally invasive procedures can be successfully utilized to prevent and arrest carious lesions. These interventions are especially effective in arresting the progress of early/incipient carious lesions. Large/cavitated lesions extending into the dentin are less amenable to current techniques due to the differences in dentin composition and structure, and caries pathology that facilitates bacterial penetration, survival and the ability of the dentin to remineralize. Furthermore, the elimination of bacteria trapped within the deeper portions of large lesions without surgical intervention poses an additional challenge. Therefore, improving the antimicrobial properties of these interventions is an effective strategy to increase their efficacy. Materials used in minimally invasive procedures are susceptible to hydrolytic and enzymatic degradation in the oral environment, resulting in a deterioration in their mechanical properties over time, increasing the propensity of interfacial bacterial ingress and biofilm formation and secondary decay and leading to treatment failure. Improving the biostability of these materials could potentially prolong the lifespan of these treatment modalities and further delay/obviate the need for surgical intervention. Hence, approaches to improve the antimicrobial and anti-degradative properties of current minimally invasive techniques should be explored to enhance their effectiveness and expand the scope of application of these interventions.

## Data Availability

Not applicable.
